# Remineralization of particulate organic carbon in an ocean oxygen minimum zone

**DOI:** 10.1038/ncomms14847

**Published:** 2017-03-21

**Authors:** E. L. Cavan, M. Trimmer, F. Shelley, R. Sanders

**Affiliations:** 1 National Oceanography Centre, European Way, Southampton SO14 3ZH, UK; 2 University of Southampton, National Oceanography Centre, European Way, Southampton SO14 3ZH, UK; 3 Queen Mary University London, Mile End Road, London E1 4NS, UK

## Abstract

Biological oceanic processes, principally the surface production, sinking and interior remineralization of organic particles, keep atmospheric CO_2_ lower than if the ocean was abiotic. The remineralization length scale (RLS, the vertical distance over which organic particle flux declines by 63%, affected by particle respiration, fragmentation and sinking rates) controls the size of this effect and is anomalously high in oxygen minimum zones (OMZ). Here we show in the Eastern Tropical North Pacific OMZ 70% of POC remineralization is due to microbial respiration, indicating that the high RLS is the result of lower particle fragmentation by zooplankton, likely due to the almost complete absence of zooplankton particle interactions in OMZ waters. Hence, the sensitivity of zooplankton to ocean oxygen concentrations can have direct implications for atmospheric carbon sequestration. Future expansion of OMZs is likely to increase biological ocean carbon storage and act as a negative feedback on climate change.

## Abstract (web summary)

The downward transfer of organic carbon from the surface to the deep ocean is increased in oxygen minimum zones relative to oxic waters. Here, the authors show reduced interactions of zooplankton with sinking particles owing to low oxygen are likely the primary reason for the observed high transfer of carbon.

It is well documented that in oxygen minimum zones (OMZs), a high fraction of the particulate organic carbon (POC) formed in the surface ocean during photosynthesis sinks to the deep ocean^1^,^2^,^3^,^4^,^5^,^6^,^7^,^8^, via the biological carbon pump^9^. Thus, remineralization length scales (RLS)^10^ that strongly influence atmospheric CO_2_ levels^11^,^12^, such that an increase in the RLS of 100 m can decrease atmospheric CO_2_ levels by ∼50 p.p.m. (ref. 11), are large in OMZs. Despite more than three decades of research the reasons for the large observed RLSs in OMZs are still unclear.

Key hypotheses invoked to explain the high RLSs observed in OMZs include: (1) changes in zooplankton behaviour^1^; (2) the sinking material being more refractory^1^; (3) preferential consumption of nitrogen-rich organic matter^2^; (4) a low oxidation rate of sinking organic matter by microbes^3^ and finally (5) the addition of material in the mesopelagic zone via chemoautotrophy^13^. OMZs have expanded over the past 50 years and are likely to expand further with global change, owing to increases in temperature and further slowing down in ventilation^14^,^15^; leading to an increase in the amount of organic carbon transferred and stored in the deep ocean. Consequently, determining what controls RLS, thus understanding the processes which control on the fate of organic particles in OMZs is important^16^.

As material sinks through the mesopelagic zone (100–1,000 m) it is subjected to a range of processes including the direct respiration by particle-associated or free-living bacteria, abiotic disaggregation^17^ and the ingestion, consumption and fragmentation to smaller, slower sinking particles by mesozooplankton^18^,^19^. Recent work suggests that in temperate, oxic waters zooplankton fragment ∼50% of the sinking particles prior to their consumption by microbes^19^. This fragmentation likely produces the observed bimodal distribution of sinking velocities with the bulk of the material either sinking fast (>100 m d^−1^) or slow (<10 m d^−1^)^20^,^21^.

To determine whether microbial turnover of POC is reduced in OMZs, giving rise to the large RLS consistently observed, we collected fast and slow-sinking particles from the Eastern Tropical North Pacific (ETNP) OMZ, along a transect from the coast to the open ocean. We measured bulk POC content and direct measurements of sinking rates to estimate POC flux profiles and RLSs. We then measured microbial oxygen consumption under non-limiting oxygen conditions on both fast and slow-sinking particles, together with their organic carbon content, to estimate their relative reactivity or carbon-specific turnover (*k*). Taking these *k* values and coupling them to our measured sinking rates we can estimate the RLS of particles sinking through the OMZ assuming microbial respiration, and ultimately remineralization, either aerobic or anaerobic^22^, was the only process involved in reducing organic carbon flux. We then compared this ‘microbial only' attenuation to POC flux attenuation, calculated from flux profiles, to estimate the relative contribution of microbial remineralization to POC turnover. Our primary results show when the observed RLS was high, microbes were responsible for ∼ 70% of the POC turnover.

## Results

### POC in an oxygen minimum zone

**Figure 1:**
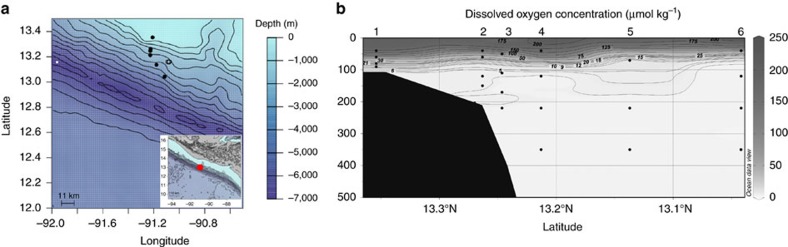
MSC deployment locations and oxygen concentrations. Marine Snow Catcher deployment locations with (**a**) Bathymetry from NOAA database, solid black circles are locations of MSCs deployed for POC flux analysis and open circles are MSC deployments for particle incubations, red circle in insert shows location of deployments in relation to the Guatemalan Pacific coast, and (**b**) Dissolved oxygen (DO) concentrations (μmol kg^−1^) to 500 m in the ETNP with latitude. Filled black area is the bathymetry. The upper oxycline occurred at ∼50 m and was slightly shallower offshore. Black dots are MSC sampling depths for POC flux analysis with the corresponding station numbers. Figure 1b was produced using Ocean Data View^67^.

Off the coast of Guatemala the ETNP OMZ (13.2 °N, 91.2 °W) had a steep upper oxycline at ∼50–70 m (Fig. 1) and a lower oxycline at ∼950 m, below which dissolved oxygen (DO) concentrations increased. By 120 m in both the onshore (bottom depth <600 m) and offshore (bottom depth>600 m) regions^23^, DO concentrations were between 2 and 5 μmol kg^−1^, with the lowest DO concentration (1.9 μmol kg^−1^, that is, the limit of detection for the Seabird sensor) at station 4, at 350 m depth.

**Figure 2:**
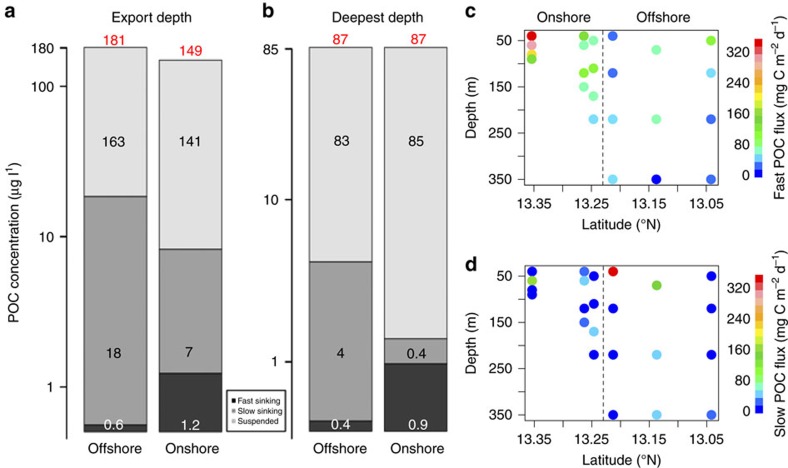
Mean POC concentrations and flux. Concentrations (μg l^−1^) are for the suspended (light-grey), slow-sinking (mid-grey) and fast-sinking (dark-grey) fractions at the (**a**) export depth (40–70 m) and (**b**) deepest depth sampled (220–350) m, black numbers are the POC concentrations of each fraction and red numbers are total POC concentration, note the log scale on the *y* axis, see text for standard errors of the mean; Fast (**c**) and slow (**d**) sinking POC fluxes (mg C m^−2^ d^−1^), the dashed line separates the onshore and offshore sampling sites.

Mean (±s.e.m.) total POC concentrations decreased by at least 62±5.6 μg l^−1^ (range 34–120 μg l^−1^) over the upper mesopelagic zone (40–350 m), with suspended particles consistently forming the largest fraction (Fig. 2a,b). Generally, concentrations of fast-sinking (∼100–200 m d^−1^) particles were lowest, apart from at the deepest deployments onshore (90–220 m) where the concentration of fast-sinking particles was 56% larger than the slow-sinking (<20 m d^−1^) fraction. This is consistent with the findings of Riley *et al*.^21^, who also deployed Marine Snow Catchers (MSCs) to measure POC fluxes and observed the majority of particles in the mesopelagic to be suspended. It has been suggested that large aggregates are hotspots for anoxic microbial activity^24^, yet we show here that suspended and slow-sinking particles are much more abundant than fast-sinking particles. Slow-sinking particles were significantly smaller (*t*=−34, d.f.=809, *P*<0.001) than fast-sinking particles, with a mean equivalent spherical diameter (ESD) of 31 μm (± 0.13 μm, s.e.m.) compared with fast sinking mean of 489 μm (±14 μm). The larger surface area to volume ratio of the smaller particles will result in more rapid colonization of free-living microbes, increasing the number of particle-associated microbes relative to organic matter, and likely result in higher remineralization rates compared with fast-sinking particles^25^.

### Particle fluxes

The flux of fast-sinking POC peaked onshore (78–350 mg C m^−2^ d^−1^ compared with 37–122 mg C m^−2^ d^−1^ offshore) and decreased with depth both onshore and offshore (Fig. 2c). Fast-sinking particles mostly comprised phytodetrital aggregates and faecal pellets, with faecal pellets being dominant at the base of the surface-mixed layer (40–70 m) and phytodetrital aggregates dominating in the upper mesopelagic zone (200–350 m).

**Figure 3:**
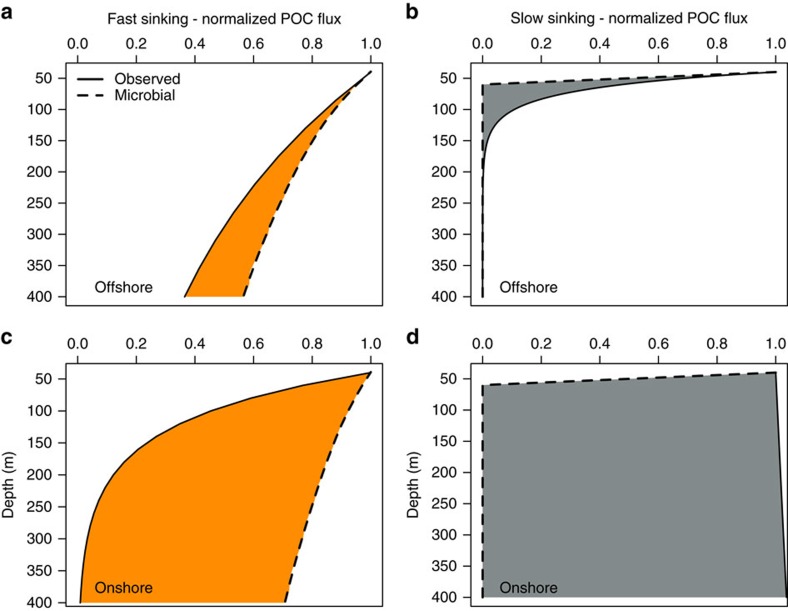
Observed and estimated microbial POC flux profiles. Observed (solid line) and predicted, assuming constant *k* estimated from microbial O_2_ uptake (dashed line), normalized POC flux profiles through the upper mesopelagic zone offshore for (**a**) fast-sinking (orange shading) and (**b**) slow-sinking particles (grey shading) and onshore for (**c**) fast-sinking and (**d**) slow-sinking particles. The shading highlights the difference between the two flux profiles, with a larger shaded area indicating a greater discrepancy between the two profiles.

The greatest attenuation of fast-sinking POC flux occurred onshore, with rapid attenuation in the upper 150 m of the water column (Fig. 3). This gave a short mean RLS (z*) of 76 m (Table 1) and a low (32%) transfer efficiency (T_*eff*_) that is, the ratio of particle flux at depth exported out of the mixed layer, was low onshore. The POC flux used to calculate T_*eff*_ was from the export depth (40–50 m) and the deepest depth sampled (220–350 m). Offshore (Fig. 3a, solid line), a higher proportion of fast-sinking POC was preserved with depth (T_*eff*_=52% and z*=357 m) and this POC flux profile was similar to others observed in OMZs^1^,^2^,^3^,^4^,^5^,^6^,^7^. An RLS of 357 m is much longer than that found in fully oxic waters of a similar temperature (*T*); the Marsay function^26^, (z*=b-a*T*) suggests that z* should be ca. 100 m, that is, closer to the z* we observed onshore of 76 m. We conclude, therefore, that the offshore environment is characterized by an efficient transfer of material through the midwater region, similar to that found in other anoxic regions, and different to that found in oxic waters.

Slow-sinking POC flux had higher maxima offshore (0–345 mg C m^−2^ d^−1^) than onshore (0–168 mg C m^−2^ d^−1^, Fig. 2d). As with the fast-sinking flux, the slow-sinking flux decreased with depth offshore (Fig. 3b, T_*eff*=_16%, z*=27 m); however, onshore, it increased at intermediate depths (Figs 2d and 3d, T_*eff*=_200%, z*=NA). This was either due to the *in situ* generation of small particles via the interior fragmentation of larger, fast-sinking particles^17^ or the resuspension of material from the seafloor. Turbidity over the sampling depths was significantly higher (*t*-test, *t*=4, df=248, *P*<0.001) onshore than offshore.

### Microbial carbon-specific turnover

**Figure 4:**
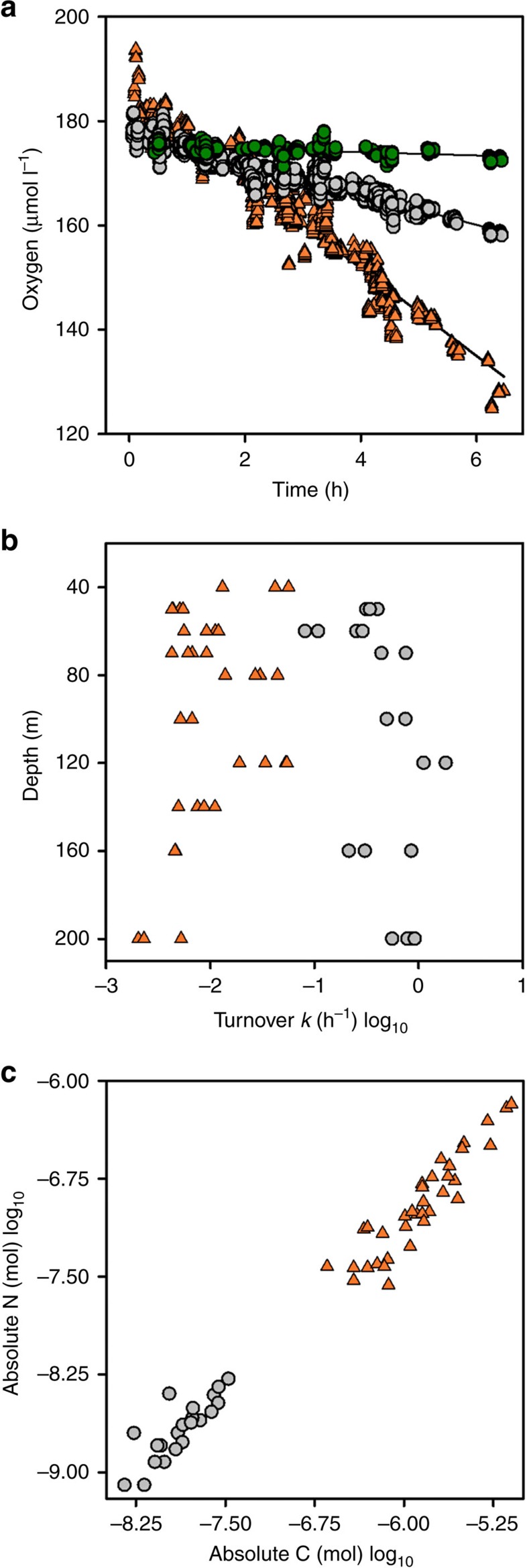
Oxygen turnover and stoichiometry of POC. (**a**) Overall estimates for change in oxygen concentration with time (Supplementary Table 2). Orange triangles and grey circles are fast and slow-sinking fractions respectively and green is the LOD (see Methods). The data are standardized to account for the random intercept fitted to each MSC deployment and incubation chamber. The slope between the two fractions is significantly different to the LOD. (**b**) *k* (Log_10_, h^−1^) calculated by normalising the oxygen uptake rates, prior to temperature corrections, by POC from (**c**) the amounts of POC and PON (mol) in each incubation chamber.

We used oxygen microelectrodes to make the first direct measurements of oxygen consumption by two fractions of sinking organic particles recovered directly from the ocean in short incubations (typically <4 h, Fig. 4a and Supplementary Fig. 1, Supplementary Table 1). We were able to detect a measurable rate of change in oxygen in 76 out of 88 incubations, from ∼25 to 7.2 μmol l^−1^ d^−1^. The water recovered with the particles from below 70 m was depleted in oxygen. To minimize any possible disaggregation of particles by reaeration we chose to measure the rate of consumption at the oxygen concentration in the recovered water and then characterize any rate limitation by oxygen. The latter proved to be minimal (Supplementary Fig. 2, Supplementary Table 2) and, more importantly, and similarly to the chamber volume, of no consequence to the final estimates of *k* (carbon-specific respiration rates, h^−1^) for either the fast or slow-sinking fractions (Supplementary Figs 3,4 and Supplementary Table 3).

We measured *k* for sinking particles between 40 and 200 m (Fig. 4b, Table 2). There is evidence of a modest change in *k,* measured at 24 °C, with depth; increasing for slow-sinking particles whereas decreasing for the fast-sinking particles (Fig. 4b, Supplementary Table 4). However, the relationship is clearly more complex than a simple linear change with depth (Supplementary Fig. 5b) and we present the more conservative interpretation of reactivity (lability) being constant with depth. Our mean (±s.e.m.) temperature-corrected fast sinking *k* estimate (*k*=0.13±0.01 d^−1^, here reported in d^−1^ to allow for comparisons) is within the reported global literature range of 0.01–0.5 d^−1^ (refs 27, 28, 29, 30, 31, 32, 33), and indistinguishable from those reported at a similar temperature by Iversen and Ploug^32^ (0.12 d^−1^).

Particles collected below 100 m would be exposed to *in situ* oxygen concentrations below 5 μmol kg^−1^ (Supplementary Fig. 6). According to Kalvelage *et al*.^34^ at oxygen concentrations of 5 μmol kg^−1^ respiration rates of the fast-sinking material (mean ESD=489 μm) would be 70–90% of those measured at elevated oxygen levels. The small size of the slow-sinking material (mean ESD=31 μm) means it is likely that respiration rates would be independent of oxygen level as diffusion limitation is unlikely to occur at even the lowest oxygen levels observed. In the OMZ core, when oxygen concentrations were below the detection limit (∼2 μmol kg^−1^), anaerobic processes would replace oxic respiration, as shown by the accumulation of nitrite after 250 m (Supplementary Fig. 6). Kamp *et al*.^35^ showed evidence of complex nitrogen transformations inside aggregates when exterior conditions were anoxic as microbes would be inside the particles too and here utilising other electron acceptors to gain energy in the core OMZ^36^.

Although field measurements of oxic respiration and nitrate reduction at the same depths indicate that potential oxygen respiration exceeds nitrate reduction and denitrification by >1 order of magnitude in OMZ waters^23^,^34^,^37^, other studies indicate that anaerobic respiration might occur at the same rate as aerobic respiration. For instance, Van Mooy *et al*.^2^ found that microbes degraded carbon at a similar rate in oxic and low oxic conditions and more recent work suggests heterotrophic bacteria biomass production and growth rates are not affected in OMZs^38^. Based on these results and the theoretical arguments outlined above based on Kalvelage *et al*.^34^ we suggest that our estimates of organic matter degradation are close to those which occurred in the low oxygen waters we sampled.

These are the first ever observations of respiration rates on slow-sinking particles; previously *k* has only been measured on large, fast-sinking particles, and often on laboratory-produced aggregates^27^,^28^,^29^,^32^. Our novel estimates of slow-sinking POC turnover were significantly higher (*t*=−5.56, df=18, *P*<0.001) than the fast-sinking particle turnover (mean temperature-corrected slow sinking *k=*5±0.4 d^−1^). There was at least an order of magnitude difference in the rate at which the two fractions are turned over by microbes (Table 2) with 99% of the carbon in the slow-sinking particles potentially being completely remineralized in less than a day, with a similar level of degradation for fast-sinking particles taking 36 days.

As previously stated that slow-sinking particles were smaller than fast-sinking particles. We believe that the elevated rates of oxygen consumption on the slow-sinking particles result from their higher surface area to volume ratio, which allow their rapid colonization by previously free-living bacteria. Oxic respiration in smaller particles is less likely to be diffusion limited than in larger fast-sinking particles and they may be may be less subjected to ballasting effects and the associated physical protection of organic material by minerals associated with larger phytoplankton^39^,^40^,^41^.

The extremely high rates of turnover, coupled to the observation that reactivity is constant with depth, suggest that slow-sinking particles in the interior are generated *in situ,* that is, they are turned over too fast and sink too slowly to be surface-derived. For instance, if a particle sinking at 5 m d^−1^ is turned over at a rate of 5 d^−1^, it would have an RLS of 1 m, so after sinking 1 m would only retain 37% (1/*e*) of its original organic carbon. The importance of slow-sinking particles in the biological carbon pump has been underestimated because of the reliance on sediment traps for collecting particles and so the origin of slow-sinking particles has been unclear^42^.

For instance, in the Joint Global Ocean Flux study sediment traps were deployed between 350 and 4,000 m, with most below 1,000 m (ref. 43), thus they would not trap small sinking particles as they are remineralised quickly in the upper ocean (Fig. 3b). This likely results in underestimates of fluxes and estimates of export efficiencies. A suggested mechanism for the production of small, slow-sinking particles is via the fragmentation of larger, fast-sinking particles^17^,^18^. Evidence in support of this can be seen in the near-perfect conservation of the POC:PON ratio (Fig. 4c) between fast and slow-sinking particles. Currently in biogeochemical models, slow-sinking particles are poorly constrained; they are only formed in the upper ocean from small phytoplankton with no additional contribution from the fragmentation of larger particles^44^,^45^, underestimating the potential contribution of slow-sinking particles to total flux.

## Discussion

If we couple these microbial turnover rates to particle sinking rates we can estimate z* (RLS) and predict an offshore profile of fast and slow-sinking POC flux attenuation, assuming only microbial respiration was degrading POC. Offshore, our predicted microbial profile closely matches that observed from our bulk particle collection POC fluxes (Fig. 3a,b). The offshore observed RLS, for fast-sinking particles, was the highest we observed (357 m, Table 1 and Fig. 3a), consistent with other OMZ particle flux studies^1^,^2^,^3^,^4^,^5^,^6^,^7^,^8^.

In this environment, our measured microbial turnover of the fast-sinking fraction (dashed line, Fig. 3a), explains ∼70% of the observed reduction in downward flux (solid line, Fig. 3a), with the remaining 30% (orange shading, Fig. 3a) presumably being due to other processes such as consumption by metazoans or fragmentation. This 70% may be an upper estimate, however decreasing *k* by 20% (in accordance with Kalvelage *et al*.^34^) still results in high microbial activity accounting for the majority of remineralisation. This situation differs considerably from that in temperate and polar oxic waters, where the main sink for fast-sinking particles is zooplankton-mediated consumption or disaggregation^19^,^46^.

Wishner *et al*.^47^ show that zooplankton (here defined as visible metazoans) are almost entirely absent from offshore OMZ waters and Williams *et al*.^48^ suggest that the few zooplankton that are there feed on surface material. We believe that these factors account for the high RLS, with the minor fraction of particle loss not attributable to microbial remineralization being caused by abiotic fragmentation^17^, rather than consumption by zooplankton. This is supported by our estimates of microbial POC attenuation for slow-sinking particles offshore (dashed line, Fig. 3b) which overestimates the POC decline with depth (grey-shaded area, Fig. 3b). As there were more particles at depth than estimated by microbial remineralization they must be being produced at a faster rate here, from the disaggregation of fast-sinking particles (orange shaded area, Fig. 3a). Fragmentation of fast-sinking particles must remove the remaining 30% of fast-sinking particles offshore as we have shown that slow-sinking particles are produced at depth from larger, fast-sinking particles and cannot persist in the mesopelagic zone (Figs 2c,d and 4b,c).

Onshore, the contribution of microbial respiration to POC flux attenuation is much lower (ca. 10%), resulting in a relatively higher contribution of particle disaggregation (orange shading, Fig. 3c) consistent with the lower RLS (Table 1). Therefore, either both zooplankton fragmentation and behaviour in the dynamic near shore environment are less impacted by low oxygen concentrations or the more energetic environment of the shelf leads to higher levels of abiotic disaggregation. In this region, the predicted slow-sinking flux did not match the observed flux well, likely due to high resuspension of slow-sinking particles from the seafloor in this upwelling region^49^.

We have shown that the lability of POC is not reduced, is constant with depth and consistent with measurements from fully oxygenated waters (rejects hypothesis 2), that nitrogen-rich material is not preferentially consumed as the POC:PON ratio was similar to the Redfield ratio^50^ at 8.3 (Fig. 4c, rejects hypothesis 3) and the potential microbial remineralization of POC is not reduced in OMZs (rejects hypothesis 4). We have rejected these three hypotheses based on what we have measured; lability (2) and stoichiometry (3). Our final assessment is that microbes in OMZ regions are responsible for up to 70% of POC remineralisation, much higher than in the temperate North Atlantic^19^. Hence, the proportion of microbial to zooplankton POC turnover is increased in OMZs and thus not reduced as suggested elsewhere (4). This leaves hypotheses 1 and 5, the changes in zooplankton behaviour or the addition of material via chemoautotrophy, as possible explanations for a high RLS in OMZs.

One chemolithoautotropic process is the use of oxidants to detoxify hydrogen sulfide resulting in carbon fixation. Schunck *et al*.^51^ observed a sulphide plume in the Eastern Tropical South Pacific (ETSP), which produced high rates of carbon fixation, up to 30% of photoautotrophic carbon fixation. However, these rates occur only if a hydrogen sulphide plume persists, which are sporadic, thus unlikely to explain the consistent pattern of high proportions of surface-produced POC through OMZs. Another chemolithoautotrophic process, anammox (anaerobic ammonium oxidation)^52^, is elevated in onshore shelf OMZs and occurs at depth-integrated (over 375 m) rates (C-conversion, Redfield ratio) of <66 mmol C m^−2^ d^−1^, but decreases rapidly offshore^23^. Therefore, anammox is also unlikely to be solely responsible for the high RLS observed offshore. Löscher *et al*.^53^ also observed that fixation of nitrogen and carbon in OMZ eddies was higher onshore in young coastal waters in the ETSP, further emphasizing that dark carbon fixation is important in coastal OMZ waters but less so offshore, so unlikely explains the high RLS.

As zooplankton are metazoans, most cannot tolerate such low (2–5 μmol kg^−1^) oxygen concentrations^36^, and so vertical migration into the mesopelagic zone is reduced in OMZs. In the coastal region, of the ETNP, zooplankton exhibit the shallowest diel vertical migration daytime depth globally of <200 m and oxygen is thought to be one of the most important factors influencing the daytime depth of migrating zooplankton^54^. A modeling study has suggested that the small population of zooplankton that have adapted to live in OMZs excrete ammonium, enhancing anammox rates, but that these exceed particle remineralization rates^55^. Zooplankton that inhabit OMZs for a portion of their day would not be able to actively feed on particles, a metabolically expensive activity. However, they could passively respire carbon and excrete ammonium at depth; although these processes in crustaceous zooplankton have been found to decrease with decreasing oxygen concentrations and, as such, estimates of diel vertical migration-mediated export rates in OMZs are thought to be unrealistically high^56^. Therefore, changes in zooplankton behaviour (hypothesis 1), mostly the reduced daytime depth of the diel vertical migrators^54^ and the low resident populations in the OMZ core^47^, are more likely to be responsible for the high proportion of surface-produced POC at depth in OMZs.

We have shown that organic matter degradation by microbes is not affected by low oxygen concentrations, as our estimates are equal to those reported from oxic waters. A study in the Chilean OMZ showed that microbial degradation rates of labile DOC are similar in oxic (>115 μmol kg^−1^) and suboxic (<22.5 μmol kg^−1^) waters^8^ and in the Peruvian margin that degradation of organic matter was not affected by oxygen^57^. As OMZs are inhospitable to metazoans, the biological community is dominated by prokaryotes and low-oxygen tolerant unicellular eukaryotes^36^,^58^. Future expansion of OMZs is expected to result in subsequent expansion of the microbial loop and a decline in trophic interactions by zooplankton^58^, hence a higher proportion of organic carbon will reach the deep oceans.

In summary, the high RLS in the offshore OMZ is most likely the result of lower particle fragmentation relative to microbial remineralization, likely owing to the absence of zooplankton in the core OMZ waters. Microbial remineralization rates in the OMZ are comparable to those in fully oxic waters but not high enough to offset the decrease in particle disaggregation and consumption by zooplankton, resulting in higher T_*eff*_ in the offshore region of the OMZ. Thus, the role of zooplankton in the biological carbon pump will become increasingly reduced with further expanding OMZs under a changing climate, leading to even higher RLSs. Numerical models suggest that a high RLS is associated with increased carbon storage, hence increased OMZ extent is likely to cause ocean biological processes to play an enhanced role in storing carbon in the ocean and drawing down atmospheric carbon dioxide. This represents a negative feedback on climate change that numerical models, which typically simulate remineralization rather poorly, need to incorporate into future structures.

## Methods

### Cruise location

Measurements were made in the ETNP on board the *RRS James Cook* from 28th December 2013 until 10th February 2014 (Fig. 1a). Particles were collected from six stations moving from onshore to offshore. Onshore stations were defined as those where the bottom depth was<600 m (stations 1–3) and offshore >600 m (ref. 23) (stations 4–6). At each station, a conductivity-temperature-depth and Niskin bottle frame was deployed with a fluorometer to identify the peak in fluorescence and a Seabird oxygen sensor to measure dissolved O_2_.

### Particle flux

Giant (300 l) MSCs (see Riley *et al*.^21^ for more information) were used to collect sinking particles from the upper mesopelagic zone (closed circles Fig. 1a). Deployment depths started from 10 m below the subsurface chlorophyll maximum (as determined from prior fluorometer casts) to 350 m water depth. After collection, the MSCs were left to settle for 2 h before being sampled. Fast-sinking particles are defined as those that after 2 h of settling are sat on the base of the MSC and collected from a base tray and slow-sinking those that contained within the base section (35 l) above the fast-sinking particles.

For fast-sinking particles, once the MSC had been emptied a tray at the bottom of the MSC containing the fast-sinking particles was removed. This base tray was taken inside the ship and any zooplankton swimmers removed using a plastic 2 ml pipette. Flakes of rust in the tray were also removed using a magnet. Photographs were systematically taken of the base tray using a Veho VMS-004 USB digital microscope at × 20 magnification. Image-J64 was used to both identify particles and measure their area and an Olympus SZX16 microscope at × 100 magnification was used to classify particles as either phytodetrital aggregates or faecal pellets. The POC mass was estimated using the same method and conversion factors^59^ as in Cavan *et al*.^60^. Fast particle sinking rates and size (ESD) were estimated using a FlowCam^61^ using *in situ* water to sink the particles through.

For slow-sinking particles, an initial 1 l sample of water was taken from the central tap on the top part of the MSC (*t*_0_) as soon as the MSC was recovered to the deck, representing a homogeneous water column. After leaving the MSCs to settle for 2 h another 1 l of water was taken from the same central tap representing the suspended fraction of particles (*t*_2_) and 1 l from the base tap representing slow-sinking and suspended particles (*b*_2_). All 1 l water samples were filtered individually onto ashed (400 °C, overnight) GF/F filters. Filters for POC were dried and stored at room temperature and acid fumed in hydrochloric acid overnight. The mass of POC was determined at Jacob's University, Germany using a CHN analyzer. To determine the mass of slow-sinking particles, the mass of POC sampled from the top (*t*_2_, suspended particles) was subtracted from the mass of POC in the base (*b*_2_, suspended+slow-sinking particles) as the base of the MSC contained both suspended and slow-sinking particles. A simplification of the SETCOL^62^ method was used to calculate the sinking rate of slow-sinking POC (equation 1):


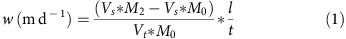


where *V*_s_ is the volume of the base of the snow catcher, *V*_t_ is the total volume of the MSC, *M*_0_ is the initial POC mass from sample *t*_0_, *M*_2_ is the POC mass of the base settled over 2 h (from sample b_2_), *l* is the length of the MSC (m) and *t* the settling duration (2 h). The SETCOL method has previously been applied to particles in MSCs in the Southern Ocean^63^, yielding slow-sinking rates of 0–3 d^−1^. The SETCOL method assumes there is a homogenous distribution of slow-sinking particles in the MSC at *t*_0_ and with using the MSC that the fast-sinking particles would have settled to the base of the MSC during the recovery to deck. Thus, any change in biomass in the MSC between *t*_0_ and *t*_2_ is due to slow-sinking particles. The fast-sinking particles would not have been sampled from the bottom tap at *t*_2_ as this is situated above the base of the MSC.

The POC flux (mg C m^−2^ d^−1^) of both fast and slow-sinking particles was calculated from the following equation^46^ (equation 2):


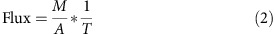


where *M* is the mass of fast or slow-sinking POC, *A* is the area of the base of the MSC (0.15 m^2^) and *T* is the sinking time calculated by dividing the length of the MSC (2 m) by the calculated fast or slow-sinking rates. To compute the observed POC flux profiles displayed in Fig. 3, the mean POC flux was taken at each depth on/offshore and for fast/slow-sinking particles and these values fitted to the exponential function of Boyd and Trull^10^ (see equation 2). All fits were significant (*P*<0.01) apart from in Fig. 3d for slow-sinking particles onshore, which, overall, increased with depth.

### Estimating microbial turnover of particulate carbon

It is important to appreciate here that our aim was not to try and make measurements representative of *in situ* particulate aerobic respiration per se but rather we used oxygen consumption, combined with measurements of organic carbon, to estimate the reactivity of that particulate carbon, that is, *k* (d^−1^). To that end, we made our measurements of oxygen consumption at non-limiting oxygen concentrations and at a constant temperature, 24 °C. To minimize any possible disaggregation of particles by reaeration we chose to measure the rate of consumption at the oxygen concentration in the recovered water and then characterize any rate limitation by oxygen. Separate MSC deployments were made (open circles Fig. 1a) between 40 and 250 m to collect particles for microbial oxygen consumption using the same protocol as above for the flux estimates. Swimmers were removed from the base tray using a plastic 2 ml pipette. In total, 400 ml of water from the base (slow-sinking and suspended particles) and the middle tap (suspended particles) were filtered through a 45-micron glass fibre filter (Whatman) for subsequent elemental analysis. We used a dedicated micro-respiration system (Unisense) with an eight-chamber rack secured in a water bath at 24 °C to hold eight glass micro-respiration chambers (4 × 4 ml and 4 × 2 ml, see below) each with a glass-coated magnetic stirrer. Stirring in the chambers was sufficient enough to mix the oxygen within the chamber and to maintain the larger particles in suspension but not violent enough to cause disaggregation (see below).

Fast-sinking particles and their associated water, from three-fourth of the base tray (1.8 l), were transferred to four chambers (two 4 ml and two 2 ml) using a Pasteur pipette (opening 4 mm) and water collected from the base of the MSC (slow-sinking particles) was placed in two chambers (one 4 ml and one 2 ml) and water from the middle tap (suspended particles) in the remaining two chambers (one 4 ml and one 2 ml). The chambers were sealed and placed in the water bath to equilibrate to temperature for 20 min in the dark before the first measurements of oxygen were made. A rapid-response, micro-oxygen electrode (OX 20, Unisense) was then lowered through the capillary in the chamber lid and data logged in each chamber for 30 s, every 30–40 min for ∼4 h.

Rates of oxygen consumption, for each fraction in each chamber, were calculated using linear regression with the lmList function in R, with any non-significant (*P*>0.05) rates of consumption being removed before continuing. We also used linear-mixed-effects-modelling^64^ to characterize the overall rates of consumption in both the fast and slow-sinking particles (slow and suspended were indistinguishable and were therefore combined), where we fitted ‘time' and ‘particle-fraction' as fixed effects, with random slopes and intercepts for both ‘chamber' and ‘MSC' where appropriate (see Supplementary Fig. 7 and Supplementary Table 5). Owing to the sampling protocol suspended and slow-sinking particles would have been present in the particle incubations for fast-sinking particles. We collected particles using pipettes and thus fast-sinking particles were incubated in *in situ* water, which would have had slower sinking and suspended particles present. Therefore, rates of consumption for the fast-sinking particles were blanked for their respective rate of consumption in their parallel slow-sinking fraction.

In total, we made 88 measurements of oxygen consumption, 12 of which had non-significant *t*-values for their slope (rate) estimates. We then used these 12 ‘non-significant' datasets in a linear-mixed-effect model to give a best estimate for our limit of detection. Accordingly, we estimated our limit of detection to be −0.31 μmol l^−1^ h^−1^ (s.e. 0.15, *t*=−1.99, *P*=0.047, Fig. 4a), which, although marginally significant, is an order of magnitude lower than our mean rate for both fractions (see Supplementary Table 1) and an order of magnitude greater than that determined with an ultrasensitive STOX sensor for raw, unadulterated, or non-concentrated, seawater of up to 0.085 μmol l^−1^ h^−1^ (ref. 65). Hence, we could only measure significant rates of oxygen consumption in seawater with particles concentrated by settling in the MSC. There was no effect of initial oxygen concentration (Supplementary Fig. 2, Supplementary Table 2), or chamber volume, on the final estimates of *k* (carbon-specific respiration rates, d^−1^) for either the fast or slow-sinking fractions (Supplementary Figs 4,5 and Supplementary Table 3).

At the end of each incubation we were able to recover all of the fast-sinking particles from each chamber and freeze them for later elemental analysis. Upon return to the United Kingdom, the fast-sinking particles were transferred directly to tin-cups, whereas the slow sinking and suspended filters were sub-sampled. The tin-cups were then combusted at 1,000 °C in an elemental analyzer (Sercon Integra 2), along with respective standards and blanks to quantify the organic carbon content. Once normalized to their respective chamber volumes, we could then estimate carbon-specific respiration constants where: *k* (h^−1^)=*μ*mol O_2_ chamber^−1^ h^−1^/*μ*mol C chamber^−1^. *K* was then converted to d^−1^ to allow comparisons with other studies but where appropriate some graphical representations and statistical analyses (Fig. 4 and Supplementary Material) were done with *k* in h^−1^. We also carefully examined the data for any chamber volume effect on either the initial raw rates of consumption or final estimates of *k* (see Supplementary Fig. 5).

### Modelling flux through the mesopelagic

To determine the extent of microbial turnover of POC in OMZs we compared the measured flux to that estimated from microbial respiration (*k*) that could be mediated by either aerobic or anaerobic respiration *in situ*. At low oxygen concentrations other electron receptors can be used for respiration such as nitrate or nitrite. Denitrification of nitrate yields a very similar Gibbs free energy to oxic respiration, followed closely by the conversion of nitrite to nitrous oxide or dinitrogen^22^. Multiple electron receptors can be used simultaneously in OMZs to maximize the free energy yield^36^. Therefore, whereas our estimates of *k* will not represent aerobic respiration in the OMZ they do represent the anoxic respiration of organic matter, which can be almost as efficient as oxic respiration. This is consolidated analysing *k* with depth (Fig. 4b) as if the exposure to oxygen was likely to have a large effect on these results then particles taken from where oxygen was at the limit of detection or absent (>120 m) in the OMZ should have a significantly higher carbon-specific respiration rate than those in the upper oxygenated waters. However, this was not observed.

We used the exponential function (as outlined by Boyd and Trull^10^) and the RLS (the exponent z*) to compare the measured POC and estimated microbial attenuation with depth (equation 3):


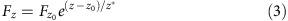


where *F*_*z*_ is the flux at depth z, *F*_*z0*_ is the shallow reference flux at depth *z*_*0*_ and z* is the RLS or the depth (m) by which 63% of the flux had been remineralized. *z*_*0*_ was determined at each station (Fig. 1, closed circles) as 10 m below the mixed layer depth, with the mixed layer depth estimated from temperature and salinity profiles using the conductivity-temperature-depth. To determine the RLS of POC, we calculated the z* exponent. To calculate z* for the measured POC fluxes we took the mean average POC flux with depth for each zone and sinking fraction and rearranged equation 3 giving z* (m). To calculate our estimated z* for microbial remineralization only we used the following equation:


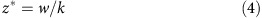


where *w* is the average calculated sinking rates (m d^−1^, equation 1, Table 2) and *k* is the microbial carbon-specific respiration, that is, turnover (d^−1^, Table 2). z* exponents were calculated for fast and slow-sinking POC fluxes both onshore and offshore (Table 1).

Both sinking rate and microbial oxygen uptake were measured at 24 °C, which is higher than the *in situ* water temperature the particles were collected from (∼10–20 °C). Therefore, we corrected for the effect of temperature. For the *k* constant we used the Q_10_ relationship^66^ where an increase in 10 °C increases respiration by a factor of 2.5, which is equivalent to an activation energy of 0.65 eV (ref. 66), and for sinking rates an increase in 9 °C results in a 40% increase in sinking rate^61^. These corrected values were used to model (equation 3) POC flux through the upper mesopelagic zone assuming microbial POC turnover was the only remineralization pathway (Fig. 3).

### Data availability

Data are available upon request from the authors. Request for materials should be addressed to E.L.C. (emma.cavan@utas.edu.au).

## Additional information

**How to cite this article:** Cavan, E. L. *et al*. Remineralization of particulate organic carbon in an ocean oxygen minimum zone. *Nat. Commun.* **8**, 14847 doi: 10.1038/ncomms14847 (2017).

**Publisher's note:** Springer Nature remains neutral with regard to jurisdictional claims in published maps and institutional affiliations.

## Supplementary Material

Supplementary Information Supplementary Figures and Supplementary Tables

## Figures and Tables

**Table 1:** POC remineralization length scales.

**Area**	**Fast-sinking particles**		**Slow-sinking particles**	
	**Observed** z* **(m)**	**Microbial** z* **(m)**	**Observed** z* **(m)**	**Microbial** z* **(m)**
Onshore	76	874	NA	0.5
Offshore	357	532	27	1.3

Mean observed and estimated, by microbial O_2_ uptake, remineralization length scales, z*'s, for fast and slow-sinking particles. NA is where there was no net attenuation of POC flux with depth, as it actually increased at intermediate depths. Offshore the microbial z* is close to that observed for the fast-sinking particles but is much higher than the observed onshore.

**Table 2:** POC sinking and turnover rates.

	**Area**	**Fast-sinking particles**		**Slow-sinking particles**	
		**Sinking rate (m** **d**^**−1**^**)**	***k*****(d**^**−1**^**)**	**Sinking rate (m** **d**^**−1**^**)**	***k*****(d**^**−1**^**)**
Initial	Onshore	171	0.35	4	13.7
	Offshore	104	0.35	9	13.7
					
Temp-corrected	Onshore	113.6	0.13	2.5	5
	Offshore	69.2	0.13	6.5	5

Initial (not corrected) and temperature-corrected mean sinking and carbon-specific respiration rates used to estimate microbial z* (Table 1). *k* estimates were made on the boundary of the on/offshore region and thus only one value is used for fast-sinking particles and one for slow-sinking particles. Applying temperature-corrections decreased sinking rates by up to 40% and *k* by 65%.
