# Supplementary material for: Remineralization of particulate organic carbon in an ocean oxygen minimum zone
Source: Nat Commun. 2017 Mar 21;8:14847. doi: 10.1038/ncomms14847 (PMC5364423; doi:10.1038/ncomms14847)
Supplement: Supplementary Information — Supplementary Figures and Supplementary Tables [file ncomms14847-s1.pdf]

Supplementary Figure 1. Raw data for change in oxygen concentration (µM) over time for both the slow and fast sinking particle fractions.

Data are from all 11 deployments of the MSC when we measured oxygen consumption. For each deployment (1-11, yellow-ribbon) we made four rate measurements (different colored symbols) with each fraction (green-ribbon). The starting concentration in oxygen varied between each set of measurements, especially for the slow fraction, but this had no consistent effect on the measured rate of change in oxygen (Fig. 2) or the final estimates of *k* measured at 24 °C (Fig. 3 and 4).

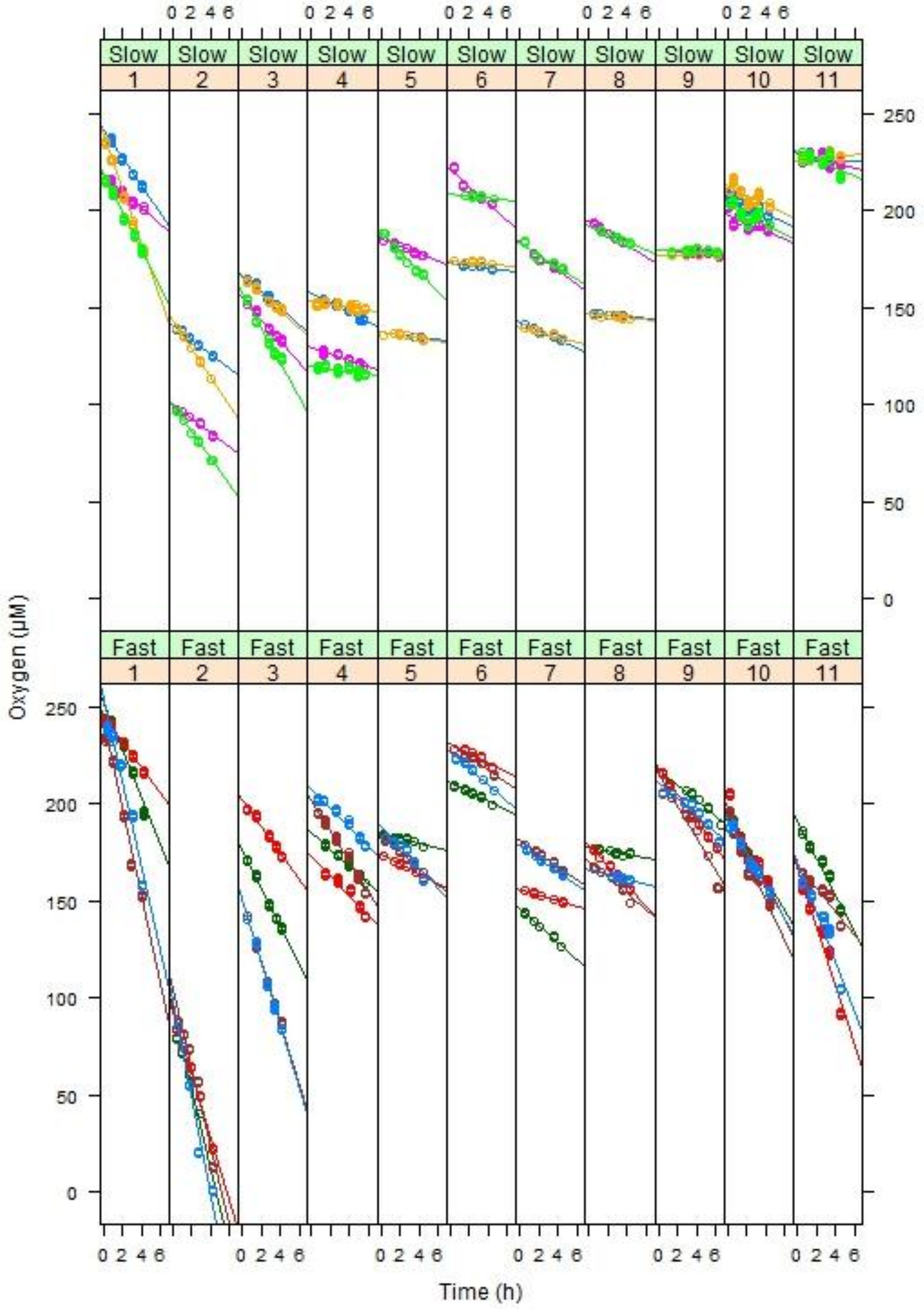

Supplementary Table 1. Output from a linear mixed-effect model.
For the overall rate of change in oxygen concentration (μM) over time for both the slow and fast sinking particle fractions. Here we model the change in oxygen as a fixed effect of both time and particle fraction with random intercepts and slopes fitted as nested random effects on each chamber within each deployment of the MSC. The rate of change in oxygen in water with the slow fraction alone is significantly slower ~35% than that with the addition of fast-sinking particles. Note, the fast rates are blanked for the inherent slow rate within each incubation in the final calculation of *k* for each fraction. The complete, original data set for each incubation can be seen in Supplementary Fig. 1.

Fixed effect	Value	s.e.	*t*-value	*P*(>|*t*|)
Intercept, μM, Fast	185.33	10.37	17.87	0.00
Slope, μM h^{-1}, Fast	-8.39	1.25	-6.68	0.00
Δ Intercept, Slow	-7.78	4.50	-1.73	0.09
Δ Slope, Slow v Fast	5.48	0.73	7.54	0.00

Supplementary Figure 2. Measured rates of change in oxygen as a function of its initial concentration.
For both the fast and slow sinking particle fractions (yellow-ribbon) for both the 2mL and 4mL incubation chambers (green-ribbon). See Table 2 below for statistical analysis.

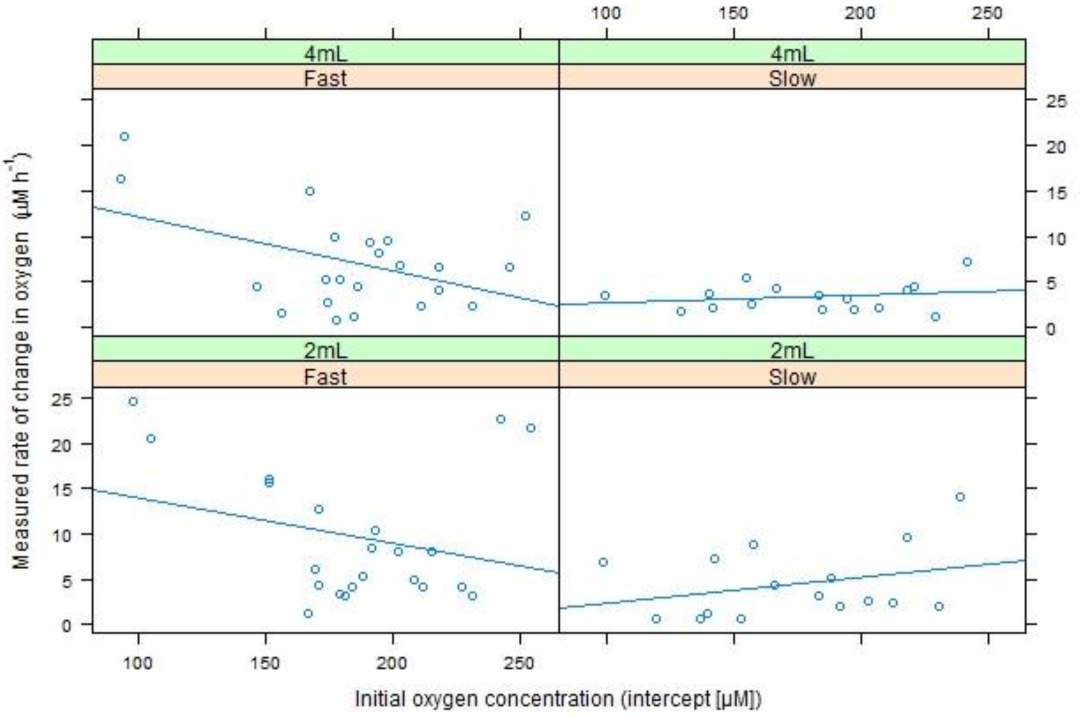

Supplementary Table 2. Effect of the initial concentration of oxygen on the estimated rate of change for both particle fractions with the 2 mL and 4 mL incubation chambers.

**Coefficients**	**Estimate**	**s.e.**	***t* value**	***P*(>|*t*|)**
Intercept, Fast, 2mL	18.82	5.37	3.50	0.00
Slope, Fast, 2mL	-0.05	0.03	-1.73	0.09
Δ Intercept, Slow	-19.41	7.83	-2.48	0.02
Δ Intercept, Fast, 4mL	-0.83	7.50	-0.11	0.91
Slope, Slow, 2mL	0.078	0.04	1.83	0.07
Δ Slope, Fast, 4mL	-0.01	0.04	-0.25	0.80
Δ Intercept, Slow, 4mL	3.25	11.16	0.29	0.77
Δ Slope, Slow, 4mL	-0.01	0.06	-0.18	0.89

Supplementary Figure 3. Estimate of *k* ($\log_{10}$) as a function of the initial concentration of oxygen.
For each measurement and particle fraction, prior to temperature corrections. No significant effect was found (Table 3).

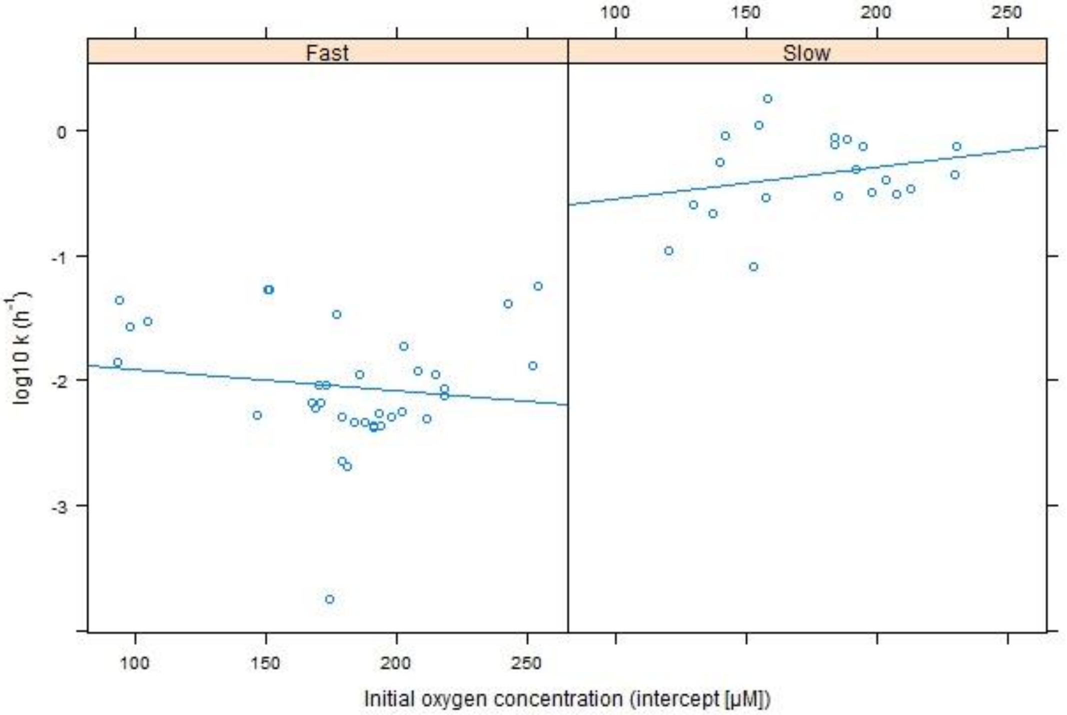

Supplementary Table 3. Confirmation that initial oxygen concentration and chamber volume had no significant effect on the overall estimate of *k* ($\log_{10}$, before

temperature corrections) for either the slow or fast sinking particles (Supplementary Fig. 4).

Coefficients	Estimate	s.e.	*t* value	*P*(>|*t*|)
**Initial Oxygen Concentration**				
Intercept (k h^{-1}) Fast	-1.74	0.35	-4.91	0.00
Slope (k h^{-1} µM^{-1}) Fast	-0.00	0.00	-0.89	0.38
Δ Intercept, Slow	0.94	0.65	1.44	0.16
Δ slope, Slow v Fast	0.00	0.00	1.19	0.24
**Chamber Volume**				
Intercept (k h^{-1}) Fast, 4mL	-2.12	0.11	-19.08	0.00
Δ Slow, 4mL	1.80	0.18	0.06	0.00
Δ Fast, 2mL	0.13	0.15	0.90	0.37
Δ Slow, 2mL	-0.19	0.25	-0.80	0.42

Supplementary Figure 4. Effect of chamber volume.
The overall effect of chamber volume (2mL versus 4mL) on the estimates of k ($\log_{10}$, before corrected for temperature) for both the fast and slow sinking particle fractions. Statistical analysis is given in Table 3.

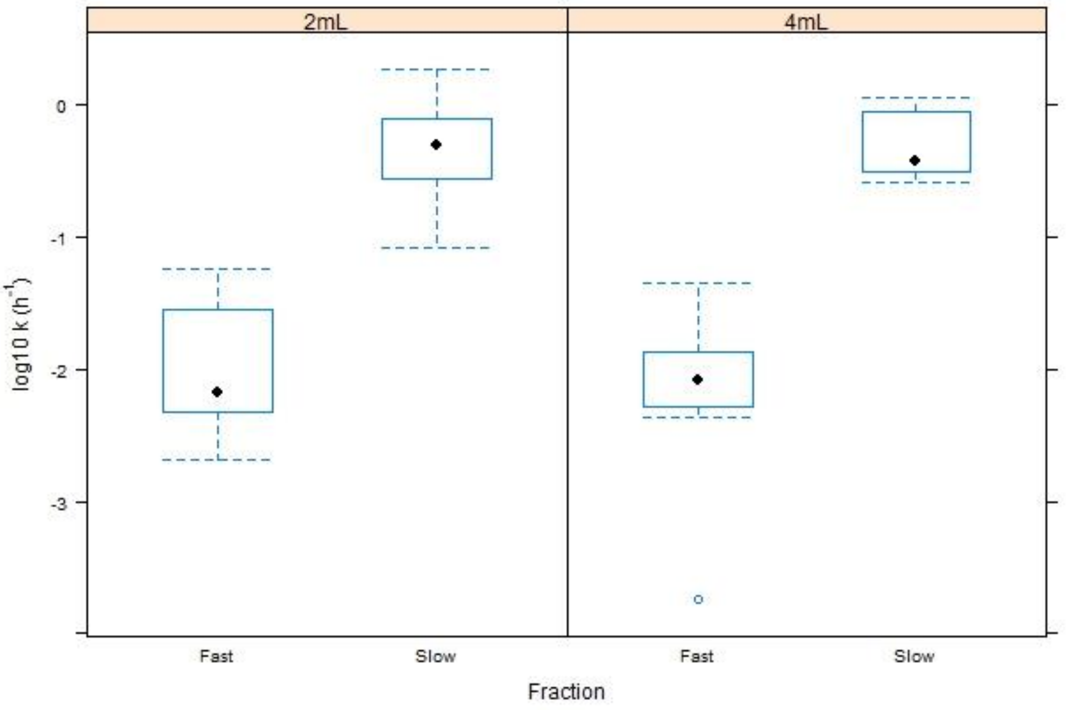

Supplementary Table 4. Analysis of covariance for the data in Supplementary Fig. 5. The effect of depth and fraction on the estimate of *k* (log_{10}) is significant in each case i.e. reactivity is different for the two fractions and also changes differently with depth for each fraction.

**Coefficients**	**Estimate**	**s.e.**	***t* value**	***P*(>|*t*|)**
Intercept (k h^{-1}) Fast	-1.67	0.16	-10.50	0.00
Depth (k h^{-1} m^{-1}) Fast	-0.00	0.00	-2.63	0.01
Δ Intercept Slow	1.01	0.25	4.03	0.00
Δ (k h^{-1} m^{-1}) Slow v Fast	0.01	0.00	3.09	0.00

Supplementary Figure 5. Carbon specific 'reactivity' turnover (*k* h^{-1} log_{10}). As a function of depth for both the fast and slow sinking particle fractions prior to temperature corrections **a**. While the overall effect of depth might be interpreted as being significant, the basic assumptions underpinning a linear model are clearly violated **b.** where there should be no obvious pattern in the residuals from the linear, ANVOCA model (Supplementary Table 4).

a.

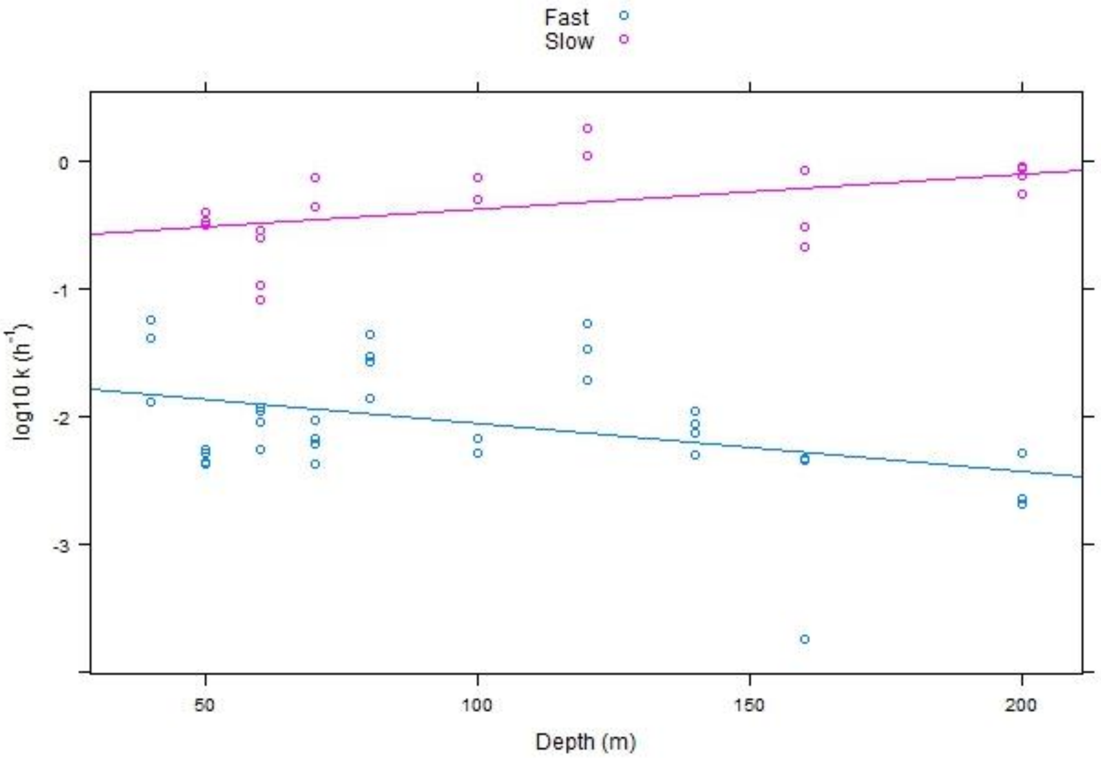

b.

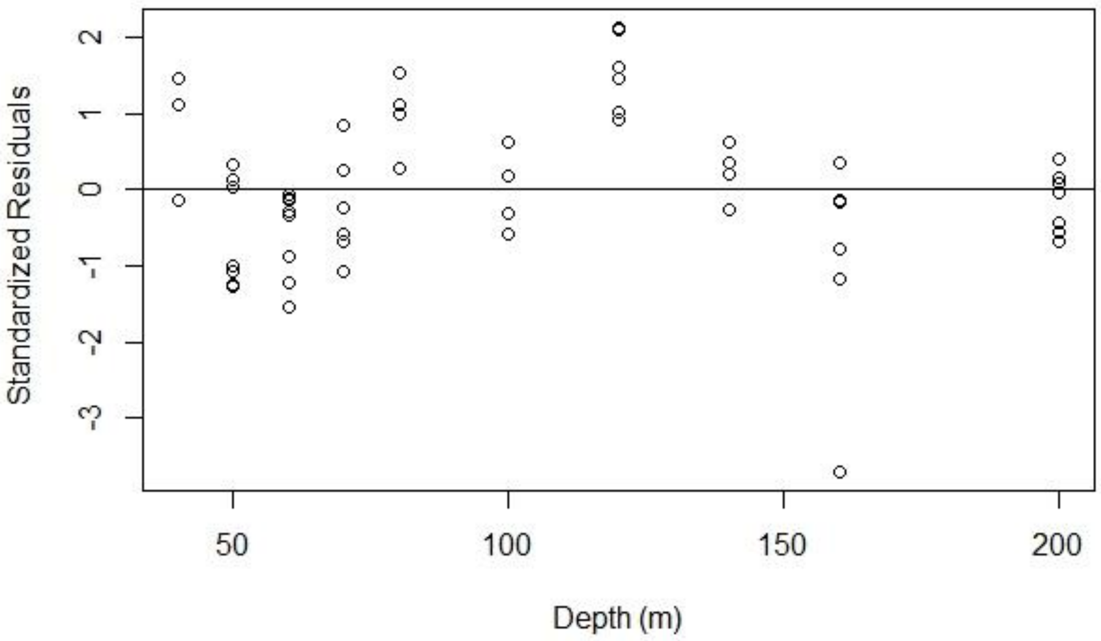

Supplementary Figure 6. Nutrient and oxygen data.
Over the upper 350 m. a) nitrate, b) nitrite, c) phosphate and d) oxygen. In d) the subset plot shows oxygen concentrations below 20 μmol L^{-1} and the flat line in oxygen concentration below 250 m.

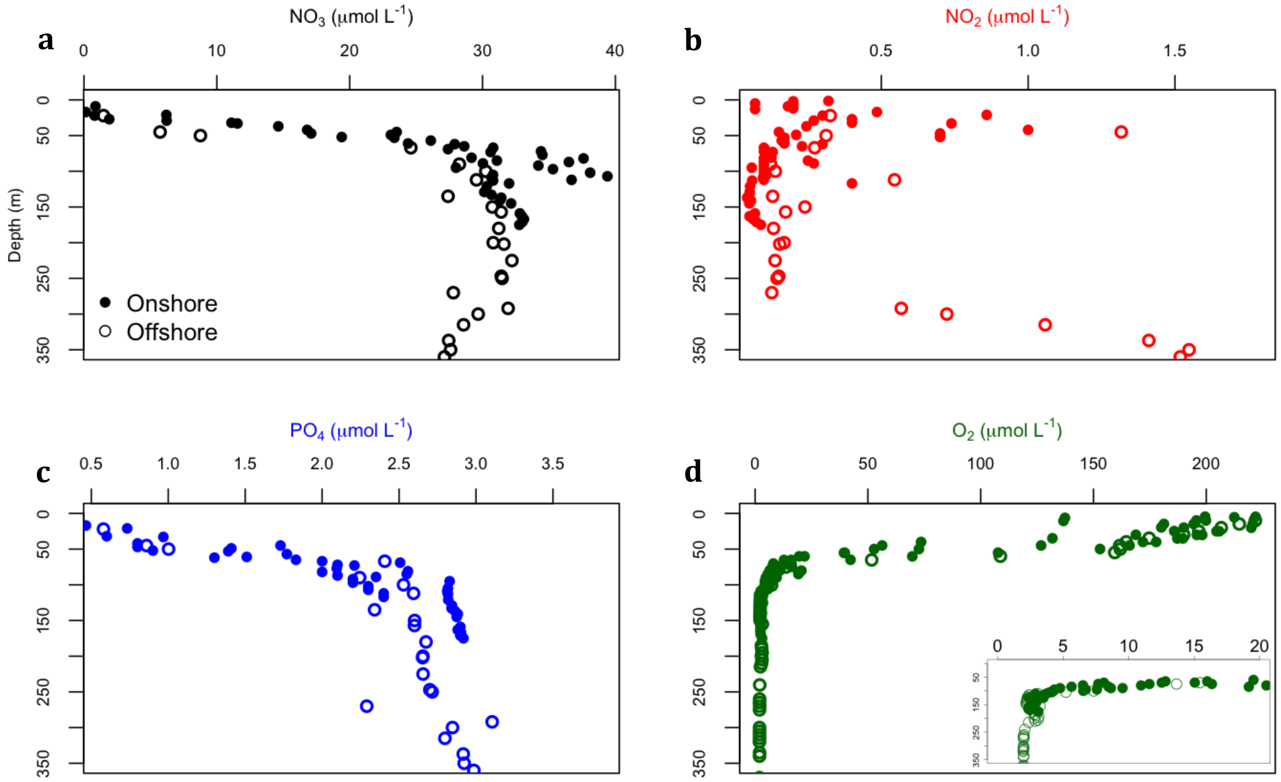

Supplementary Figure 7. Change in oxygen concentration.

All the raw data for change in oxygen concentration (µM) as a function of time for both the slow and fast sinking particle fractions (as in Supplementary Fig. 1) with all data from the 11 catchers combined for either the 2 mL or 4 mL chambers. With the fast sinking, visible aggregates, the ratio of organic carbon to total oxygen (mol) is higher in the 2 mL chambers compared to the 4 mL and the rate of consumption was faster, on average (Supplementary Table 5 below). For the slow sinking fraction the ratio is constant between chamber volumes and no such effect is apparent. Lines are for illustration only and are not part of linear mixed effects analysis for the overall fixed effects (fraction + chamber volume).

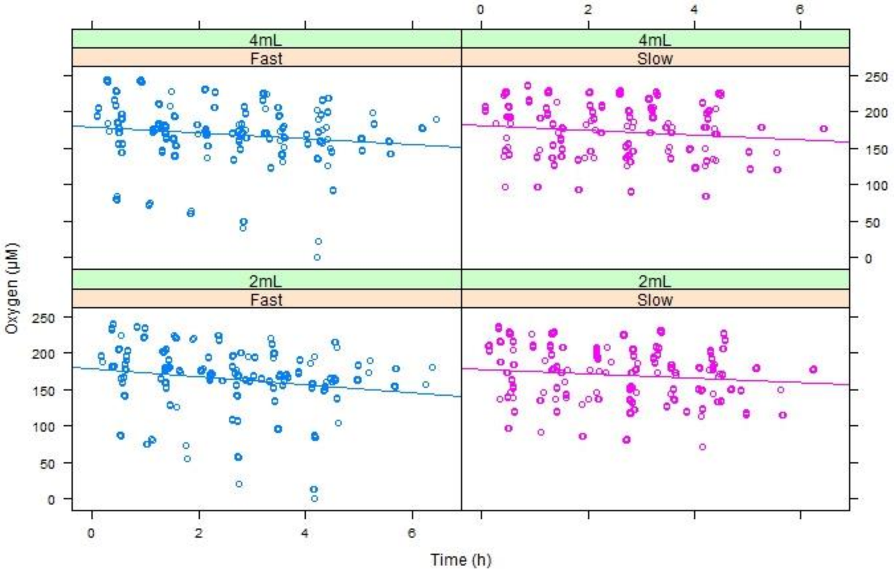

Supplementary Table 5. The overall effect of chamber volume (2 mL versus 4 mL) on the raw rate of change in oxygen concentration for the fast and slow sinking particles. Output is from a linear mixed-effects model where we fitted 'Time (h)' and 'chamber volume (mL)' as fixed effects and included random slopes and intercepts for each of the 11 deployments of the MSC. For simplicity, the coefficients are estimated separately for the fast and slow fractions. As described above, the rate of change (µM h^{-1}) was more rapid for the fast fraction in the 2 mL chambers versus 4 mL* but this was not the case for the slow fraction†. Once normalized, however, to chamber volume and total organic carbon per chamber this artifact was removed (Supplementary Fig 4 and Supplementary Table 3).

**"Fast"**	**Estimate**	**s.e.**	***t* value**	***P*(>|*t*|)**
Intercept (µM)	186.79	11.73	15.92	0.00
rate (µM h^{-1})	-7.84	1.76	-4.44	0.00
Δ intercept 2mL vs 4mL	-2.48	1.22	-2.03	0.04
Δ rate 2mL vs 4mL	-1.06	0.40	-2.62	0.09*

**"Slow"**	**Estimate**	**s.e.**	***t* value**	***P*(>|*t*|)**
Intercept (µM)	179.56	10.40	17.26	0.00
rate (µM h^{-1})	-3.09	0.87	-3.55	0.00
Δ intercept 2mL vs 4mL	-4.04	1.42	-2.85	0.00
Δ rate 2mL vs 4mL	0.51	0.46	1.09	0.28†
